# Experienced disrespect & abuse during childbirth and associated birth characteristics: a cross-sectional survey in the Netherlands

**DOI:** 10.1186/s12884-024-06360-y

**Published:** 2024-02-29

**Authors:** Denise R. Leijerzapf, Marit S. G. van der Pijl, Martine H. Hollander, Elselijn Kingma, Ank de Jonge, Corine J. M. Verhoeven

**Affiliations:** 1 Amsterdam UMC Location Vrije Universiteit Amsterdam, Midwifery Science, Amsterdam, Netherlands; 2 Midwifery Academy Amsterdam Groningen, InHolland, Amsterdam, Netherlands; 3 Amsterdam Public Health, Quality of Care, Amsterdam, the Netherlands; 4 Department of Primary and Long-Term Care, University of Groningen, University Medical Center Groningen, Groningen, the Netherlands; 5 Amalia Children’s Hospital, Department of Obstetrics, Radboud University Medical Center, Nijmegen, the Netherlands; 6 Department of Philosophy, King’s College London, London, UK; 7 Amsterdam Reproduction & Development Research Institute, Amsterdam, the Netherlands; 8 Division of Midwifery, School of Health Sciences, University of Nottingham, Nottingham, UK; 9 Department of Obstetrics and Gynaecology, Maxima Medical Centre, Veldhoven, the Netherlands

**Keywords:** Disrespect and abuse, Mistreatment, Obstetric violence, Respectful maternity care, Patient-provider interaction, Informed consent, Communication, Support, Birth experience

## Abstract

**Background:**

Experiencing upsetting disrespect and abuse (D&A) during labour and birth negatively affects women’s birth experiences. Knowing in what circumstances of birth women experience upsetting situations of D&A can create general awareness and help healthcare providers judge the need for extra attention in their care to help reduce these experiences. However, little is known about how different birth characteristics relate to the experience of D&A. Previous studies showed differences in birth experiences and experienced D&A between primiparous and multiparous women. This study explores, stratified for parity, (1) how often D&A are experienced in the Netherlands and are considered upsetting, and (2) which birth characteristics are associated with these upsetting experiences of D&A.

**Methods:**

For this cross-sectional study, an online questionnaire was set up and disseminated among women over 16 years of age who gave birth in the Netherlands between 2015 and 2020. D&A was divided into seven categories: emotional pressure, unfriendly behaviour/verbal abuse, use of force/physical violence, communication issues, lack of support, lack of consent and discrimination. Stratified for parity, univariable and multivariable logistic regression analyses were performed to examine which birth characteristics were associated with the upsetting experiences of different categories of D&A.

**Results:**

Of all 11,520 women included in this study, 45.1% of primiparous and 27.0% of multiparous women reported at least one upsetting experience of D&A. Lack of consent was reported most frequently, followed by communication issues. For both primiparous and multiparous women, especially transfer from midwife-led to obstetrician-led care, giving birth in a hospital, assisted vaginal birth, and unplanned cesarean section were important factors that increased the odds of experiencing upsetting situations of D&A. Among primiparous women, the use of medical pain relief was also associated with upsetting experiences of D&A.

**Conclusion:**

A significant number of women experience upsetting disrespectful and abusive care during birth, particularly when medical interventions are needed after the onset of labour, when care is transferred during birth, and when birth takes place in a hospital. This study emphasizes the need for improving quality of verbal and non-verbal communication, support and adequate decision-making and consent procedures, especially before, during, and after the situations of birth that are associated with D&A.

**Supplementary Information:**

The online version contains supplementary material available at 10.1186/s12884-024-06360-y.

## Background

In maternity care, the main goal is to optimize health outcomes for mother and child. In recent years, women’s birth experience has become an important indicator of good quality care [1]. The World Health Organization (WHO) states that the clinical management of maternity care is well understood, but more priority should be given to women’s experiences [2]. A negative or traumatic childbirth experience has short- and long-term impact on a woman’s emotional wellbeing, sometimes even leading to postpartum depression or post-traumatic stress disorder [3, 4]. It also may affect bonding between mother and child [3, 4].

Studies in high income countries such as Sweden and Canada found that respectively 6.8% and 9.3% of women experienced childbirth as negative [5, 6]. Personal characteristics such as age, parity, and social status, and birth characteristics such as mode of birth, pain relief, medical interventions, duration of birth, and environment are factors that may be associated with women’s experiences [7–10]. Continuous support throughout birth is also associated with more positive experiences [8]. A questionnaire conducted among Dutch women with a self-reported traumatic birth experience revealed that lack of control, lack of support and communication issues were important factors in their experience [11]. In 2016 in the Netherlands, a social media campaign #breakthesilence was organized, in which women shared their negative experiences with maternity care. A content analysis of this campaign revealed that ineffective communication, lack of consent and loss of autonomy were the most common reasons for a negative or traumatic experience [12]. This indicates that factors related to patient-provider interaction play an important role in birth experience.

A study in the Netherlands using four domains of the ReproQ questionnaire (based on the WHO responsiveness model) showed that most postpartum women are positive about patient-provider interaction in maternity care (mean score above 3.5 in a scale 1–4 for every domain) [13, 14]. However, not all women experience this interaction as pleasant or respectful [13]. Disrespectful or abusive care plays an important role in experiencing birth as negative or traumatic [15–17]. Disrespect and abuse (D&A) during labour and birth, are defined as follows: ‘*interactions or facility conditions that local consensus deem to be humiliating or undignified, and those interactions or conditions that are experienced as or intended to be humiliating or undignified’* [18]*.* The terms ‘obstetric violence’ [16, 19] and ‘mistreatment’ [15] are also used to describe similar phenomena. No term is ideal. The term ‘obstetric violence’ is often understood as implying that care providers intentionally harm their patients, and ‘mistreatment’ seems to downplay the seriousness of the problem [20, 21]. Although the term ‘disrespect and abuse’ can be difficult to operationalize, it is also used by the WHO and therefore a commonly used term [20, 22]. According to Bohren et al. (2015), D&A during childbirth can be divided into seven categories: ‘physical abuse, sexual abuse, verbal abuse, stigma and discrimination, failure to meet professional standards of care, poor rapport between women and providers, and health system conditions and constraints’ [15].

In low- and middle-income countries, D&A during labour and birth are found to be common, with reported percentages from 33.3% up to 75.7% [23–26]. A study from Germany shows that over 77% of women report at least one form of mistreatment, indicating that D&A also take place in high income countries [27]. Non-consented care is a common category in high income countries, next to lack of information, being forced into medical acts, violation of physical privacy, dismissing birth plans, and verbal abuse and neglect [27–29]. First analyses of a large survey on D&A among women who gave birth in the Netherlands show that lack of choices and lack of communication are most often reported (39.8% and 29.9% respectively) [17]. Since D&A can be experienced differently by every woman, results were further specified into whether women experienced these situations as upsetting or not. Over 90% of women who had no upsetting experience of D&A rated their birth as (very) positive [17]. The more upsetting experiences of D&A women had, the more likely they were to rate their overall birth experience as very negative or traumatic [17]. Parity and migrant background were important personal factors in the upsetting experience of D&A [17]. For all categories of D&A, multiparous women had lower odds of upsetting D&A compared to primiparous (adjusted odds ratio (AOR) ranging between 0.47 and 0.65) [17].

Previous studies on D&A showed how often D&A was experienced (as upsetting) and what personal characteristics were associated with upsetting D&A, but not what circumstances of birth were associated with (upsetting) D&A [17]. Since the experience of D&A often affects the overall birth experience, research on birth characteristics associated with overall birth experience might provide some insight [12, 17]. However, studies focusing on birth characteristics associated with (individual categories of) D&A have not yet been performed. Known differences in birth characteristics and experiences of birth and D&A between primiparous and multiparous women, substantiate the importance of stratification [17, 30]. Knowing what circumstances of birth are associated with experienced D&A can create general awareness and help healthcare providers judge the need for extra attention in their care to help reduce these experiences. This contributes to the improvement of experienced care, an important element of quality of care [1].

This study explores, stratified for parity, (1) how often D&A are experienced in the Netherlands and are considered upsetting, and (2) which birth characteristics are associated with these upsetting experiences of D&A.

## Methods

### Study setting

In the Netherlands, independent community midwives care for women with low risk of complications throughout pregnancy, birth, and the postpartum period (midwife-led care). Midwife-led labour and birth can take place at home or in a birth centre or hospital (institution), based on the preference of the pregnant woman. If a complication or risk factor occurs during pregnancy or birth, transfer to a hospital where care is provided by clinical midwives and (resident) obstetricians, supervised by an obstetrician (obstetrician-led care) is recommended [31]. Of all births in 2021 in the Netherlands, 14% took place at home, 13% were institutional births in midwife-led care, and 73% took place in obstetrician-led care in the hospital [32].

### Measurement tool and data collection

This cross-sectional study uses data of an online survey conducted in the Netherlands. More detailed description of the measurement tool and data collection are described by van der Pijl et al. (2022) [17]. In short: an online questionnaire, available in both English and Dutch, was disseminated, consisting of five subsections: 1) birth characteristics, 2) D&A during birth, 3) verbal consent, 4) overall birth experience, and 5) personal characteristics. Based on existing literature and considering the Dutch context, questions about D&A were divided into seven categories: emotional pressure, unfriendly behaviour/verbal abuse, use of force/physical violence, communication issues, lack of support, lack of consent and discrimination [12, 15, 33]. D&A were investigated by asking respondents whether they had experienced a specific situation and, if they did, whether they experienced this as upsetting [yes/no]. The composition of questions about D&A aim to gain insight into both verbal and non-verbal communication. An overview of the questions and categories of D&A can be found in Supplement 1.

Data were collected between October 26 and December 17, 2020. The questionnaire was disseminated via social media channels, with help of social media influencers, and professional and client organizations such as midwifery practices and organizations supporting single moms or migrant women [17].

### Ethical considerations and privacy

Official ethical approval by the Medical Research Ethics Committee (MREC) was sought, but not required (MREC, Amsterdam UMC, no. 2020.084, 14 April 2020). By voluntarily filling out the questionnaire, women gave consent to participate. To maintain privacy, IP addresses were excluded from data export, email addresses were saved in a separate database, only birth month and year of the baby were asked, and zip codes only gave information about the region of residence. Data were stored on a secured file (managed by the IT department of Amsterdam UMC) for a maximum of 15 years and only authorized persons have access to it.

### Study population

All women over 16 years of age who gave birth in the Netherlands were able to participate, regardless of their gestational age or what kind of birth experience they had. Women were asked to answer questions about their most recent birth. Women who did not give birth in the five years prior to completing the questionnaire (2015–2020) or did not fill out all questions about their birth characteristics or D&A were excluded.

### Dependent and independent variables

Baseline characteristics (age, ethnic background, level of education, marital status at time of birth, singleton or multiple pregnancy, and gestational age) were categorized according to the Centraal Bureau Statistiek (Central Bureau of Statistics, CBS) classification or relevance [34, 35]. Before or during COVID-19 pandemic was dichotomized based on the first national restrictions [36]. Birth characteristics (onset of labour, healthcare provider throughout birth, medical pain relief, place of birth, and mode of birth) were categorized by relevance. An overview of how variables were categorized is shown in Supplement 2.

All separate questions of D&A were dichotomized into ‘have not experienced it OR did not consider it upsetting’ and ‘experienced AND considered upsetting’. If a woman had experienced at least one upsetting situation of D&A in a category, she was assigned to the group ‘experienced and considered upsetting’ for that category. Data are presented for all seven categories of D&A separately. Also, a composite outcome of all seven categories of D&A was made to compare no upsetting experience of D&A at all with the upsetting experience of at least one situation in any category of D&A (‘any category of disrespect and abuse’).

### Data analysis

All analyses were performed stratified for parity due to known differences in birth characteristics and experiences of birth and D&A between primiparous and multiparous women [17, 30]. Descriptive statistics were used to describe baseline and birth characteristics, and (upsetting) experiences of all categories of D&A. Pearson’s Chi-Square test was used to determine whether characteristics differed between primiparous and multiparous women. *P*-values < 0.05 were considered to be statistically significant.

Associations between birth characteristics and the upsetting experiences of different categories of D&A were determined using bivariable logistic regression analyses. To adjust for confounding, baseline and birth characteristics were added into a multivariable logistic regression model, including age, ethnic background, level of education, marital status, singleton or multiple pregnancy, gestational age, before or during COVID-19 pandemic, onset of labour, healthcare provider throughout birth, pain relief, place of birth, and mode of birth. Based on previous research, categories with known or expected lowest odds of an upsetting experience of D&A were chosen as reference category [7–10, 17]. Multicollinearity between birth characteristics was checked by measuring the variance inflation factor (VIF) [37]. Odds ratios (OR) and adjusted odds ratios (AOR) with 95% confidence intervals (CI) were obtained. All statistical analyses were performed using SPSS, version 27 [38].

## Results

**Fig. 1:**
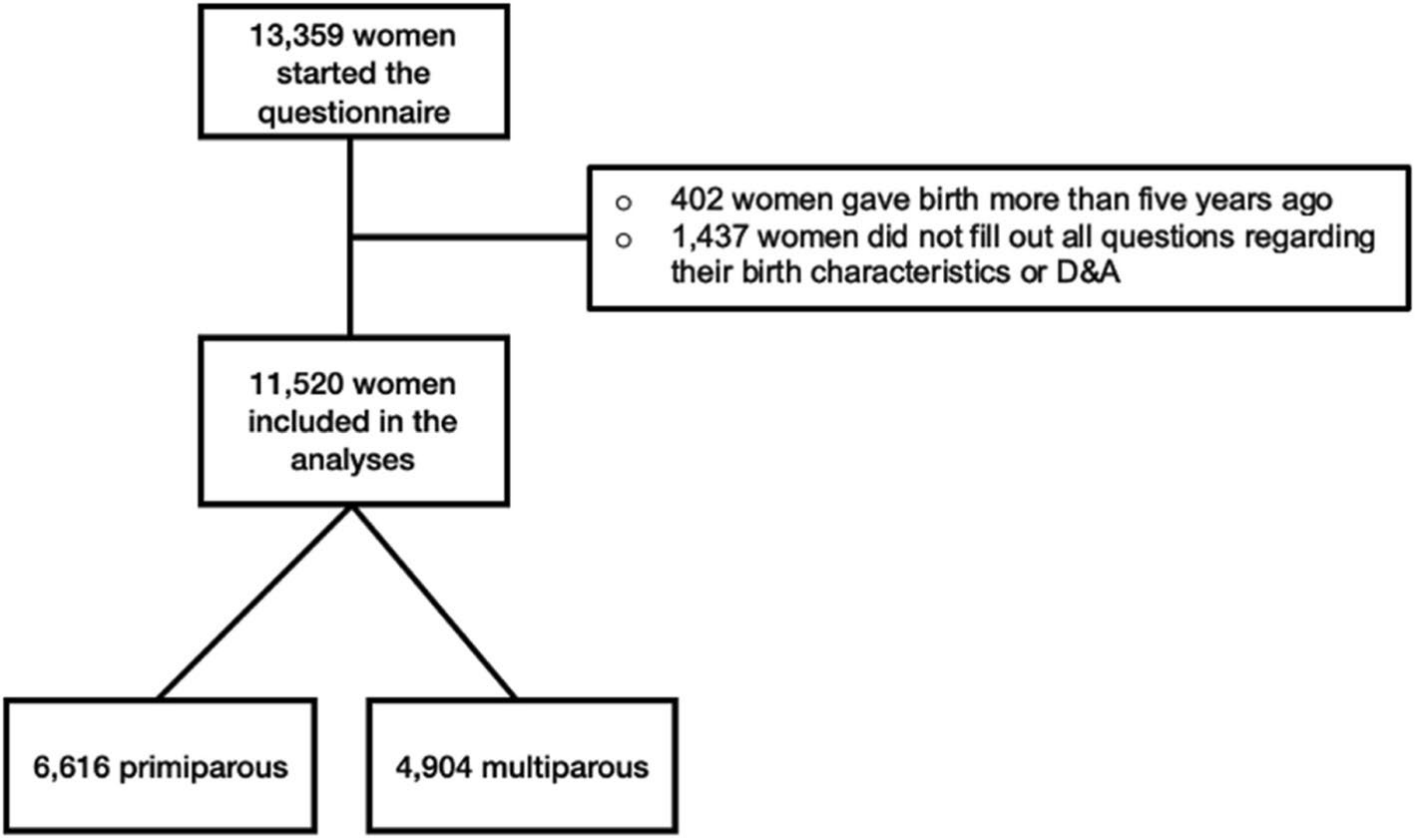
Flowchart of the study population

Of all 13,359 women who started the questionnaire, 11,520 were included in the analyses, of whom 6,616 (57.4%) were primiparous and 4,904 (42.6%) were multiparous (Fig. 1). Table 1 shows the respondents’ baseline and birth characteristics, stratified for parity. For the comparison of the similar study population with Dutch national statistics, see van der Pijl et al. (2023) [39].

**Table 1:** Baseline and birth characteristics of the study population (*n* = 11,520)

		**Total n (%)**	**Primiparous n (%)**	**Multiparous n (%)**	***P*-value**
**Total**		11520 (100)	6616 (57.4)	4904 (42.6)	
**Maternal age**	< 25 years	1052 (9.6)	861 (13.6)	191 (4.1)	** < 0.001**
	25–35 years	8771 (79.8)	5064 (79.7)	3707 (79.9)	
	> 35 years	1170 (10.6)	425 (6.7)	745 (16.0)	
**Ethnic background**	Dutch	9438 (83.0)	5429 (83.2)	4009 (82.7)	**0.02**
	Western	504 (4.4)	311 (4.8)	193 (4.0)	
	Non-western	1430 (12.6)	785 (12.0)	645 (13.3)	
**Level of education**	Low	673 (6.1)	389 (6.1)	284 (6.1)	1.00
	Middle	2659 (24.3)	1537 (24.3)	1122 (24.3)	
	High	7617 (69.6)	4402 (69.6)	3215 (69.6)	
**Marital Status at time of birth**	Married	6535 (59.4)	3459 (54.5)	3076 (66.3)	** < 0.001**
	Living together	4171 (37.9)	2669 (42.0)	1502 (32.3)	
	Living apart together	122 (1.1)	96 (1.5)	26 (0.6)	
	Single	169 (1.6)	130 (2.0)	39 (0.8)	
**Singleton or multiple pregnancy**	Singleton	11297 (98.1)	6486 (98.0)	4811 (98.1)	0.84
	Multiple	223 (1.9)	130 (2.0)	93 (1.9)	
**Gestational age**	Preterm (< 37 weeks)	669 (5.8)	447 (6.8)	222 (4.5)	** < 0.001**
	Term (37–41 + 6 weeks)	10448 (90.9)	5895 (89.2)	4553 (93.1)	
	Postterm (≥ 42 weeks)	381 (3.3)	264 (4.0)	117 (2.4)	
**Before or during COVID-19 pandemic**	Prior COVID-19 pandemic	7521 (65.3)	4237 (64.0)	3284 (67.0)	** < 0.001**
	During COVID-19 pandemic	3999 (34.7)	2379 (36.0)	1620 (33.0)	
**Onset of labour**	Spontaneous	7759 (67.3)	4383 (66.3)	3376 (68.8)	** < 0.001**
	Induction	3108 (27.0)	1960 (29.6)	1148 (23.4)	
	C-section	653 (5.7)	273 (4.1)	380 (7.8)	
**Healthcare provider throughout birth**	Midwife-led care	3899 (33.9)	1558 (23.6)	2341 (47.7)	** < 0.001**
	Transfer of care^a^	3090 (26.8)	2422 (36.6)	668 (13.6)	
	Obstetrician-led care	4531 (39.3)	2636 (39.8)	1895 (38.7)	
**Pain relief during labour**	None	6946 (64.2)	3296 (52.2)	3650 (81.1)	** < 0.001**
	Epidural analgesia	2425 (22.4)	2017 (32.0)	408 (9.1)	
	Remifentanil	1184 (11.0)	813 (12.9)	371 (8.2)	
	Other^b^	256 (2.4)	183 (2.9)	73 (1.6)	
**Place of birth**	Home	2400 (20.8)	868 (13.1)	1532 (31.2)	** < 0.001**
	Midwife-led institutional^c^	1796 (15.6)	844 (12.8)	952 (19.4)	
	Obstetrician-led hospital^d^	7324 (63.6)	4904 (74.1)	2420 (49.4)	
**Mode of birth**	Spontaneous	7248 (62.9)	3368 (50.9)	3880 (79.1)	** < 0.001**
	Planned C-section	590 (5.1)	237 (3.6)	353 (7.2)	
	Spontaneous with episiotomy	1369 (11.9)	1058 (16.0)	311 (6.3)	
	Assisted vaginal birth	1066 (9.3)	965 (14.6)	101 (2.1)	
	Unplanned C-section	1247 (10.8)	988 (14.9)	259 (5.3)	

*P*-value conducted using Pearson’s Chi-Square test

*C-section *Caesarean section

^a^Transfer from midwife-led care to obstetrician-led care

^b^Sterile water injection, Entonox or pethidine

^c^Birth centre or hospital with community midwife

^d^Hospital with hospital-based care provider (medical indication)

### (Upsetting) experiences of D&A

At least one upsetting experience in any category of D&A was reported by 45.1% of primiparous and 27.0% of multiparous women (Table 2). Both primiparous and multiparous women reported lack of consent most frequently, with 26.7% and 15.1% respectively reporting upsetting lack of consent. Communication issues were second most often experienced as upsetting (26.5% for primiparous and 13.6% for multiparous women). (Upsetting) experiences of emotional pressure and of discrimination were reported least often in both groups. For all categories except lack of consent, most women considered their experience as upsetting.

**Table 2:** Experienced disrespect and abuse, presented per category and stratified for parity (*n* = 11,520)

	**Primiparous (*n* = 6616)**			**Multiparous (*n* = 4904)**		
	**Not experienced n (%)**	**Experienced, but not upsetting n (%)**	**Experienced and upsetting n (%)**	**Not experienced n (%)**	**Experienced, but not upsetting n (%)**	**Experienced and upsetting n (%)**
**Emotional pressure**	6385 (96.5)	26 (0.4)	205 (3.1)	4788 (97.6)	20 (0.4)	96 (2.0)
**Unfriendly behaviour / verbal abuse**	5833 (88.2)	112 (1.7)	671 (10.1)	4566 (93.1)	59 (1.2)	279 (5.7)
**Use of force / physical violence**	4958 (74.9)	620 (9.4)	1038 (15.7)	4202 (85.7)	259 (5.3)	443 (9.0)
**Communication issues**	4203 (63.5)	660 (10.0)	1753 (26.5)	3911 (79.8)	324 (6.6)	669 (13.6)
**Lack of support**	4900 (74.1)	213 (3.2)	1503 (22.7)	4179 (85.2)	138 (2.8)	587 (12.0)
**Lack of consent**	2688 (40.6)	2163 (32.7)	1765 (26.7)	3129 (63.8)	1034 (21.1)	741 (15.1)
**Discrimination**	6548 (99.0)	8 (0.1)	60 (0.9)	4878 (99.4)	3 (0.1)	23 (0.5)
**Any category of disrespect and abuse**	1993 (30.1)	1640 (24.8)	2983 (45.1)	2562 (52.2)	1019 (20.8)	1323 (27.0)

### Associations between birth characteristics and D&A

Results of univariable and multivariable analyses to examine which birth characteristics were associated with upsetting experiences of different categories of D&A are presented in Tables 3 and 4 (primiparous women) and 4 (multiparous women). For the category discrimination, groups were too small to calculate AORs. Frequencies and percentages of upsetting experiences of the D&A categories for all birth characteristics can be found in Supplement 3.

**Table 3:** Birth characteristics associated with upsetting experiences of disrespect and abuse among primiparous women (*n* = 6,616)

		**Any category of disrespect and abuse**		**Emotional pressure**		**Unfriendly behaviour/ verbal abuse**		**Use of force / physical violence**		**Communication issues**		**Lack of support**		**Lack of consent**		**Discrimination**
		*OR (95% CI)*	*AOR (95% CI)*	*OR (95% CI)*	*AOR (95% CI)*	*OR (95% CI)*	*AOR (95% CI)*	*OR (95% CI)*	*AOR (95% CI)*	*OR (95% CI)*	*AOR (95% CI)*	*OR (95% CI)*	*AOR (95% CI)*	*OR (95% CI)*	*AOR (95% CI)*	*OR (95% CI)*
**Onset of labour**	Spontaneous	Ref	Ref	Ref	Ref	Ref	Ref	Ref	Ref	Ref	Ref	Ref	Ref	Ref	Ref	Ref
	Induction	*1.80* *(1.61 – 2.00)*	1.15 (0.98 – 1.34)	*1.55* *(1.16 – 2.07)*	1.06 (0.70 – 1.60)	*1.41* *(1.19 – 1.66)*	0.94 (0.74 – 1.19)	*1.63* *(1.42 – 1.88)*	1.23 (1.00 – 1.50)	*1.51* *(1.34 – 1.69)*	1.05 (0.89 – 1.25)	*1.44* *(1.27 – 1.63)*	0.95 (0.80 – 1.14)	*1.74* *(1.55 – 1.95)*	**1.23** **(1.04 – 1.46)**	*1.81* *(1.07 – 3.08)*
	C-section	1.09 (0.85 – 1.40)	1.23 (0.56 – 2.71)	1.10 (0.53 – 2.28)	0.87 (0.10 – 7.46)	1.09 (0.72 – 1.64)	1.44 (0.32 – 6.43)	*0.54* *(0.35 – 0.48)*	1.26 (0.27 – 5.81)	0.95 (0.71 – 1.26)	0.92 (0.37 – 2.27)	*1.36* *(1.03 – 1.80)*	1.76 (0.65 – 4.81)	*0.67* *(0.49 – 0.93)*	6.34 (0.83 – 48.47)	2.09 (0.73 – 5.96)
**Healthcare provider throughout birth**	Midwife-led care	Ref	Ref	Ref	Ref	Ref	Ref	Ref	Ref	Ref	Ref	Ref	Ref	Ref	Ref	Ref
	Transfer of care^a^	*4.65* *(4.01 – 5.40)*	**1.56** **(1.21 – 2.02)**	*3.34* *(1.94 – 5.72)*	**2.60** **(1.06 – 6.38)**	*4.84* *(3.52 – 6.67)*	1.59 (0.96 – 2.64)	*3.69* *(3.69 – 6.12)*	**1.77** **(1.17 – 2.68)**	*4.73* *(3.91 – 5.72)*	**1.59** **(1.16 – 2.18)**	*4.01* *(3.29 – 4.90)*	**1.42** **(1.02 – 1.97)**	*4.29* *(3.56 – 5.17)*	**1.44** **(1.05 – 1.97)**	*14.93* *(2.01 – 110.65)*
	Obstetrician-led care	*4.68* *(4.04 – 5.43)*	**1.44** **(1.09 – 1.89)**	*4.12* *(2.43 – 6.99)*	**2.71** **(1.06 – 6.93)**	*4.66* *(3.39 – 6.41)*	1.47 (0.87 – 2.50)	*4.56* *(3.55 – 5.87)*	**1.56** **(1.01 – 2.41)**	*4.38* *(3.62 – 5.29)*	1.36 (0.97 – 1.90)	*4.09* *(3.36 – 4.99)*	1.34 (0.95 – 1.91)	*4.17* *(3.46 – 5.02)*	1.26 (0.90 – 1.78)	*21.56* *(2.95 – 157.40)*
**Pain relief during labour**	None	Ref	Ref	Ref	Ref	Ref	Ref	Ref	Ref	Ref	Ref	Ref	Ref	Ref	Ref	Ref
	Epidural	*2.52* *(2.25 – 2.82)*	**1.23** **(1.06 – 1.41)**	*1.66* *(1.21 – 2.28)*	0.89 (0.61 – 1.29)	*2.33* *(1.93 – 2.80)*	**1.32** **(1.06 – 1.63)**	*1.89* *(1.62 – 2.20)*	0.96 (0.80 – 1.15)	*2.30* *(2.02 – 2.60)*	**1.22** **(1.04 – 1.42)**	*2.10* *(1.84 – 2.40)*	**1.20** **(1.02 – 1.41)**	*2.10* *(1.85 – 2.38)*	1.07 (0.92 – 1.24)	*3.31* *(1.81 – 6.04)*
	Remifentanil	*2.71* *(2.32 – 3.18)*	**1.51** **(1.26 – 1.81)**	*1.78* *(1.18 – 2.68)*	1.16 (0.74 – 1.80)	*2.03* *(1.59 – 2.60)*	1.29 (0.99 – 1.69)	*2.46* *(2.03 – 2.98)*	**1.43** **(1.15 – 1.77)**	*2.45* *(2.08 – 2.90)*	**1.49** **(1.23 – 1.80)**	*2.14* *(1.80 – 2.56)*	**1.32** **(1.08 – 1.61)**	*2.31* *(1.96 – 2.73)*	**1.38** **(1.14 – 1.66)**	1.78 (0.73 – 4.34)
	Other^b^	*2.25* *(1.67 – 3.04)*	**1.45** **(1.05 – 2.02)**	0.91 (0.33 -2.51)	0.72 (0.26 – 2.01)	*2.29* *(1.48 – 3.55)*	1.55 (0.97 – 3.09)	*1.74* *(1.18 – 2.56)*	1.12 (0.74 – 1.77)	*2.43* *(1.78 – 3.33)*	**1.60** **(1.14 – 2.26)**	*2.56* *(1.86 – 3.53)*	**1.70** **(1.20 – 2.40)**	1.36 (0.96 – 1.92)	0.89 (0.61 – 1.28)	1.13 (0.15 – 8.54)
**Place of birth**	Home	Ref	Ref	Ref	Ref	Ref	Ref	Ref	Ref	Ref	Ref	Ref	Ref	Ref	Ref	Ref
	Midwife-led institutional^c^	*1.86* *(1.46 – 2.37)*	**1.58** **(1.22 – 2.05)**	1.68 (0.69 – 4.08)	1.09 (0.39 – 3.06)	1.74 (0.96 – 3.15)	1.43 (0.78 – 2.65)	1.31 (0.85 – 2.02)	1.09 (0.68 – 1.74)	*2.17* *(1.54 – 3.05)*	**1.88** **(1.30 – 2.70)**	*2.22* *(1.55 – 3.18)*	**1.81** **(1.23 – 2.64)**	*1.82* *(1.31 – 2.52)*	**1.62** **(1.14 – 2.31)**	2.06 (0.19 – 22.75)
	Obstetrician-led hospital^d^	*6.65* *(5.47 – 8.08)*	**2.92** **(2.13 – 4.01)**	*4.19* *(2.06 – 8.54)*	1.78 (0.58 – 5.47)	*6.87* *(4.28 – 11.04)*	**3.10** **(1.58 – 6.09)**	*5.11* *(3.68 – 7.10)*	**2.07** **(1.23 – 3.47)**	*7.09* *(5.36 – 9.38)*	**3.12** **(2.05 – 4.74)**	*6.54* *(4.86 – 8.81)*	**3.35** **(2.17 – 5.20)**	*6.17* *(4.74 – 8.03)*	**3.32** **(2.21 – 4.97)**	*10.20* *(1.41 – 73.73)*
**Mode of birth**	Spontaneous	Ref	Ref	Ref	Ref	Ref	Ref	Ref	Ref	Ref	Ref	Ref	Ref	Ref	Ref	Ref
	Planned C-section	1.17 (0.89 – 1.53)	1.15 (0.62 – 2.12)	1.16 (0.53 – 2.54)	3.97 (0.45 – 35.24)	1.24 (0.77 – 2.00)	1.20 (0.48 – 2.95)	0.66 (0.39 – 1.13)	1.11 (0.43 – 2.87)	1.17 (0.84 – 1.63)	**2.08** **(1.05 – 4.12)**	*1.53* *(1.12 – 2.10)*	1.33 (0.69 – 2.59)	*0.66* *(0.45 – 0.97)*	0.98 (0.47 – 2.06)	2.26 (0.66 – 7.69)
	Spontaneous with episiotomy	*1.90* *(1.65 – 2.18)*	**1.43** **(1.22 – 1.67)**	1.42 (0.96 – 2.10)	1.19 (0.79 – 1.79)	*1.71* *(1.35 – 2.15)*	**1.31** **(1.03 – 1.68)**	*2.38* *(1.97 – 2.89)*	**1.94** **(1.58 – 2.38)**	*1.84* *(1.57 – 2.16)*	**1.44** **(1.21 – 1.72)**	*1.61* *(1.36 – 1.90)*	**1.25** **(1.04 – 1.49)**	*1.95* *(1.67 – 2.28)*	**1.54** **(1.30 – 1.83)**	*2.54* *(1.28 – 5.01)*
	Assisted vaginal birth	*3.06* *(2.64– 3.54)*	**1.82** **(1.54 – 2.15)**	1.06 (0.68 – 1.65)	0.61 (0.37 – 1.01)	*2.28* *(1.83 – 2.85)*	**1.37** **(1.07 – 1.75)**	*3.84* *(3.20—4.61)*	**2.52** **(2.06 – 3.09)**	*2.86* *(2.44 – 3.34)*	**1.84** **(1.54 – 2.20)**	*2.23* *(1.90 – 2.63)*	**1.42** **(1.18 – 1.70)**	*2.94* *(2.52 – 3.43)*	**1.84** **(1.55 – 2.18)**	1.29 (0.54 – 3.07)
	Unplanned C-section	*3.51* *(3.02 – 4.07)*	**2.07** **(1.74 – 2.46)**	*1.95* *(1.36 – 2.80)*	1.43 (0.96 – 2.15)	*2.58* *(2.08 – 3.20)*	**1.56** **(1.23 – 2.00)**	*3.01* *(2.49 – 3.63)*	**2.06** **(1.66 – 2.55)**	*3.61* *(3.10 – 4.21)*	**2.52** **(2.11 – 3.01)**	*2.70* *(2.30 – 3.16)*	**1.76** **(1.47 – 2.12)**	*2.83* *(2.42 – 3.30)*	**1.88** **(1.58 – 2.25)**	*2.90* *(1.49 – 5.66)*

For some of the comparisons, groups were too small to calculate AORs

*OR* Odds ratio, *AOR* Adjusted odds ratio, *CI* Confidence interval, *C-section* caesarean section

^a^Transfer from midwife-led care to obstetrician-led care

^b^Sterile water injection, Entonox or pethidine

^c^Birth centre or hospital with community midwife

^d^Hospital with hospital-based care provider (medical indication)

**Table 4:** Birth characteristics associated with upsetting experiences of disrespect and abuse among multiparous women (*n* = 4,904)

		**Any category of disrespect and abuse**		**Emotional pressure**		**Unfriendly behaviour/ verbal abuse**		**Use of force/ physical violence**		**Communication issues**		**Lack of support**		**Lack of consent**		**Discrimination**
		*OR (95% CI)*	*AOR (95% CI)*	*OR (95% CI)*	*AOR (95% CI)*	*OR (95% CI)*	*AOR (95% CI)*	*OR (95% CI)*	*AOR (95% CI)*	*OR (95% CI)*	*AOR (95% CI)*	*OR (95% CI)*	*AOR (95% CI)*	*OR (95% CI)*	*AOR (95% CI)*	*OR (95% CI)*
**Onset of labour**	Spontaneous	Ref	Ref	Ref	Ref	Ref	Ref	Ref	Ref	Ref	Ref	Ref	Ref	Ref	Ref	Ref
	Induction	*2.27 (1.97 – 2.62)*	1.09 (0.90 – 1.32)	1.39 (0.89 – 2.16)	0.88 (0.51 – 1.52)	*2.06 (1.59 – 2.68)*	0.97 (0.70 – 1.35)	*1.87 (1.51 – 2.30)*	0.94 (0.71 – 1.22)	*2.06 (1.72 – 2.46)*	1.01 (0.80 – 1.28)	*2.19 (1.81 – 2.64)*	1.13 (0.88 – 1.44)	*2.19 (1.85 – 2.59)*	1.15 (0.92 – 1.44)	1.88 (0.73 – 4.85)
	C-section	*1.68 (1.33 – 2.11)*	0.91 (0.34 – 2.45)	0.71 (0.29 – 1.78)	-	*1.63 (1.07 – 2.50)*	0.39 (0.10 – 1.46)	1.03 (0.70 – 1.53)	**-**	*2.03 (1.54 – 2.67)*	1.45 (0.40 – 5.32)	*2.06 (1.55 – 2.76)*	0.85 (0.27 – 2.72)	*0.65 (0.45 – 0.94)*	0.55 (0.15 – 2.05)	*4.08 (1.41 – 11.80)*
**Healthcare provider throughout birth**	Midwife-led care	Ref	Ref	Ref	Ref	Ref	Ref	Ref	Ref	Ref	Ref	Ref	Ref	Ref	Ref	Ref
	Transfer of care^a^	*3.90 (3.21 – 4.74)*	**1.94 (1.45 – 2.60)**	*5.20 (2.95 – 9.14)*	1.34 (0.50 – 3.56)	*5.44 (3.66 – 8.07)*	**2.26 (1.17 – 4.35)**	*5.84 (4.31 – 7.92)*	**2.57 (1.58 – 4.16)**	*4.80 (3.72 – 6.19)*	**1.74 (1.16 – 2.62)**	*3.67 (2.81 – 4.86)*	**1.93 (1.26 – 2.94)**	*4.24 (3.36 – 5.36)*	**2.04 (1.42 – 2.93)**	2.764 (0.59 – 11.81)
	Obstetrician-led care	*3.97 (3.42 – 4.61)*	**1.67 (1.22 – 2.28)**	*2.69 (1.60 – 4.53)*	0.65 (0.24 – 1.77)	*5.21 (3.72 – 7.30)*	1.73 (0.88 – 3.42)	*4.47 (3.44 – 5.81)*	**1.80 (1.08 – 3.00)**	*4.44 (3.60 – 5.47)*	1.34 (0.87 – 2.05)	*4.22 (3.40 – 5.23)*	**1.83 (1.18 – 2.86)**	*3.34 (2.76 – 4.04)*	1.45 (0.99 – 2.13)	*4.98 (1.66 – 14.91)*
**Pain relief during labour**	None	Ref	Ref	Ref	Ref	Ref	Ref	Ref	Ref	Ref	Ref	Ref	Ref	Ref	Ref	Ref
	Epidural	*2.89 (2.34 – 3.56)*	1.12 (0.88 – 1.44)	1.75 (0.94 – 3.28)	0.72 (0.35 – 1.48)	*3.16 (2.24 – 4.45)*	1.24 (0.83 – 1.85)	*2.53 (1.89 – 3.38)*	1.05 (0.75 – 1.47)	*2.58 (2.01 – 3.32)*	0.94 (0.70 – 1.27)	*2.67 (2.06 – 3.47)*	1.25 (0.92 – 1.71)	*2.45 (1.93 – 3.11)*	0.97 (0.73 – 1.28)	2.45 (0.68 – 8.82)
	Remifentanil	*2.29 (1.83 – 2.86)*	0.97 (0.75 – 1.25)	*2.10 (1.14 – 3.86)*	0.86 (0.44 – 1.69)	*2.86 (1.98 – 4.13)*	1.14 (0.75 – 1.73)	*2.94 (2.20 – 3.93)*	1.25 (0.90 – 1.74)	*2.57 (1.98 – 3.34)*	1.05 (0.78 – 1.42)	*2.58 (1.96 – 3.40)*	1.37 (1.00 – 1.87)	*2.13 (1.65 – 2.75)*	0.93 (0.69 – 1.24)	2.70 (0.75 – 9.71)
	Other^b^	*3.74 (2.35 – 5.96)*	**2.30 (1.37 – 3.85)**	*3.36 (1.19 – 9.48)*	1.62 (0.48 – 5.62)	*5.13 (2.75 – 9.55)*	**2.86 (1.40 – 5.80)**	*3.06 (1.68 – 5.55)*	1.41 (0.70 – 2.83)	*2.95 (1.73 – 5.03)*	1.54 (0.84 – 2.83)	*2.99 (1.72 – 5.21)*	1.75 (0.94 – 3.25)	*3.90 (2.40 – 6.33)*	**2.27 (1.31 – 3.94)**	4.60 (0.59 – 36.06)
**Place of birth**	Home	Ref	Ref	Ref	Ref	Ref	Ref	Ref	Ref	Ref	Ref	Ref	Ref	Ref	Ref	Ref
	Midwife-led institutional^c^	*1.56 (1.25 – 1.96)*	**1.37 (1.08 – 1.75)**	0.94 (0.37 – 2.39)	0.82 (0.27 – 2.48)	1.78 (0.99 – 3.18)	1.29 (0.67 – 2.49)	1.22 (0.79 – 1.89)	1.01 (0.64 – 1.61)	1.38 (0.98 – 1.94)	1.25 (0.87 – 1.81)	*1.99 (1.41 – 2.80)*	**1.73 (1.19 – 2.52)**	*1.48 (1.10 – 1.99)*	1.29 (0.93 – 1.77)	1.61 (0.23 – 11.45)
	Obstetrician-led hospital^d^	*4.78 (4.02 – 5.68)*	**2.55 (1.83 – 3.55)**	*4.16 (2.26 – 7.67)*	**7.87 (2.57 – 24.06)**	*7.31 (4.70 – 11.38)*	**3.80 (1.79 – 8.06)**	*5.24 (3.86 – 7.11)*	**2.66 (1.55 – 4.57)**	*5.46 (4.25 – 7.02)*	**3.37 (2.13 – 5.34)**	*5.20 (3.97 – 6.81)*	**2.38 (1.47 – 3.88)**	*4.23 (3.38 – 5.29)*	**2.69 (1.79 – 4.04)**	*6.05 (1.41 – 26.03)*
**Mode of birth**	Spontaneous	Ref	Ref	Ref	Ref	Ref	Ref	Ref	Ref	Ref	Ref	Ref	Ref	Ref	Ref	Ref
	Planned C-section	*1.42 (1.12 – 1.80)*	0.96 (0.48 – 2.00)	0.47 (0.15 – 1.51)	0.37 (0.05 – 2.84)	1.40 (0.89 – 2.21)	0.56 (0.19 – 1.66)	0.98 (0.65 – 1.49)	2.34 (0.62 – 8.81)	*1.74 (1.29 – 2.33)*	1.12 (0.51 – 2.46)	*1.77 (1.31 – 2.39)*	1.03 (0.44 – 2.41)	*0.58 (0.40 – 0.86)*	1.12 (0.42 – 3.01)	*3.41 (1.11 – 10.51)*
	Spontaneous with episiotomy	*1.61 (1.286– 2.07)*	1.09 (0.83 – 1.4)	1.09 (0.47 – 2.52)	0.63 (0.24 – 1.60)	1.29 (0.79 – 2.13)	0.82 (0.48 – 1.40)	1.32 (0.89 – 1.96)	0.81 (0.53 – 1.25)	*1.52 (1.10 – 2.10)*	1.05 (0.74 – 1.48)	1.37 (0.97 – 1.93)	0.92 (0.64 – 1.34)	*1.59 (1.19 – 2.14)*	1.15 (0.84 – 1.58)	-
	Assisted vaginal birth	*5.25 (3.49 – 7.89)*	**2.55 (1.63 – 3.99)**	*2.88 (1.14 – 7.29)*	1.18 (0.41 – 3.45)	*4.88 (2.90 – 8.21)*	**2.26 (1.27 – 4.01)**	*5.48 (3.53 – 8.51)*	**2.42 (1.49 – 3.95)**	*4.80 (3.16 – 7.29)*	**2.25 (1.42 – 3.57)**	*2.65 (1.65 – 4.28)*	1.29 (0.76 – 2.19)	*4.83 (3.22 – 7.25)*	**2.72 (1.74 – 4.24)**	2.98 (0.39 – 22.96)
	Unplanned C-section	*4.62 (3.57– 5.98)*	**2.23 (1.62 – 3.08)**	*2.92 (1.59 – 5.35)*	1.77 (0.85 – 3.67)	*4.31 (3.01 – 6.16)*	**1.66 (1.04 – 2.64)**	*4.23 (3.12 – 5.74)*	**2.43 (1.68 – 3.52)**	*5.75 (4.40 – 7.52)*	**2.91 (2.08 – 4.07)**	*3.81 (2.86 – 5.07)*	**1.73 (1.20 – 2.49)**	*3.59 (2.74 – 4.71)*	**2.21 (1.58 – 3.08)**	*5.86 (2.07 – 16.55)*

For some of the comparisons, groups were too small to calculate AORs

*OR* Odds ratio, *AOR* Adjusted odds ratio, *CI* Confidence interval, *C-section* Caesarean section

^a^Transfer from midwife-led care to obstetrician-led care

^b^Sterile water injection, Entonox or pethidine

^c^Birth centre or hospital with community midwife

^d^Hospital with hospital-based care provider (medical indication)

#### Primiparous women

For primiparous women, all birth characteristics were associated with the upsetting experience of D&A. Women reported more upsetting lack of consent after induction of labour compared to spontaneous onset of labour (AOR 1.23). Compared to midwife-led care throughout birth, transfer from midwife-led to obstetrician-led care increased the odds of upsetting experiences in all categories of D&A except for unfriendly behaviour/verbal abuse (AOR ranged from 1.42 to 2.60). Women who received obstetrician-led care throughout birth reported upsetting situations of emotional pressure and use of force/physical violence more frequent than women who received midwife-led care throughout birth (AOR 2.71 and 1.56). Upsetting situations of communication issues and lack of support were more often reported by women who used any method of medical pain relief (epidural, remifentanil or other), compared to women who used no pain relief (AOR ranged from 1.20 to 1.70). Additionally, the use of remifentanil was associated with higher odds of experiencing upsetting use of force/physical violence and lack of consent, and the use of epidural analgesia with unfriendly behaviour/verbal abuse. Compared to home birth, giving birth in an institution (midwife-led) increased the odds of upsetting experiences of communication issues, lack of support and lack of consent (AOR ranged from 1.62 to 1.88). Compared to home birth, hospital birth (obstetrician-led) was associated with higher odds of upsetting experiences of D&A in all categories except for emotional pressure (AOR ranged from 2.07 to 3.35 with the highest odds for lack of support and lack of consent). Compared to spontaneous mode of birth, spontaneous birth with episiotomy, assisted vaginal birth and unplanned C-section increased the odds of upsetting experiences of D&A in almost all categories of D&A (AOR ranged from 1.25 to 2.52, with the highest odds for use of force/physical violence and communication issues). Planned C-section only increased the odds of experiencing upsetting communication issues (AOR 2.08).

#### Multiparous women

For multiparous women, the characteristics healthcare provider throughout birth, pain relief during labour, place of birth, and mode of birth were associated with the experience of at least one upsetting situation of D&A. Onset of labour was not associated with upsetting experiences of D&A. Compared to midwife-led care, transfer from midwife-led to obstetrician-led care showed increased odds of upsetting D&A in all categories except for emotional pressure (AOR ranged from 1.74 to 2.57). Compared to midwife-led care, obstetrician-led care increased the odds of experiencing upsetting use of force/physical violence and lack of support (AOR 1.80 and 1.83). The use of epidural analgesia and remifentanil were not associated with upsetting D&A. However, compared to no pain relief, other methods of pain relief (sterile water injection, Entonox or pethidine) were associated with higher odds of upsetting unfriendly behaviour/verbal abuse and lack of consent (AOR 2.86 and 2.27). Compared to home birth, birth in an institution (midwife-led) increased the odds of experiencing upsetting lack of support (AOR 1.73). Birth in a hospital (obstetrician-led) increased the odds of upsetting experiences in all categories of D&A (AOR ranged from 2.66 to 7.87, with highest odds for emotional pressure). Assisted vaginal birth and unplanned C-section increased the odds of upsetting experiences in almost all categories of D&A, compared to spontaneous mode of birth. For unplanned C-section, AOR ranged from 1.66 to 2.91, with the highest odds for communication issues. For assisted vaginal birth, AOR ranged from 2.25 to 2.72, with the highest odds for lack of consent.

## Discussion

Of all 11,520 women included in this study, 45.1% of primiparous and 27.0% of multiparous women reported at least one upsetting experience in any category of D&A. Both primiparous and multiparous women reported lack of consent most frequently, followed by communication issues.

For both primiparous and multiparous women, healthcare provider throughout birth, place and mode of birth were important characteristics in the experience of D&A. Compared to spontaneous birth, assisted vaginal birth and unplanned C-section increased the odds of upsetting D&A in almost all categories in both groups. Also transfer from midwife-led to obstetrician-led care increased the odds of upsetting experiences of D&A in almost all categories for both groups. Compared to home birth, birth in an institution (midwife-led) or hospital (obstetrician-led) were associated with more upsetting experiences of D&A for both groups. Among primiparous women, the use of epidural analgesia or remifentanil was associated with upsetting experiences of most D&A categories, whilst this was not found among multiparous women. Only among primiparous women, planned C-section was associated with upsetting experiences of communication issues, and induction of labour with lack of consent, compared to spontaneous mode and onset of birth.

### Interpretation

Our results show that upsetting experiences of D&A during birth are often reported among women who gave birth in the Netherlands. Of all categories of D&A, lack of consent was reported most often (as upsetting), followed by communication issues. This is consistent with the results of previous studies from high income countries [27–29].

Our results show that in general, upsetting situations of D&A were experienced more often among women who needed medical interventions. This is in line with findings of previous research [5, 40, 41]. An explanation can be that situations where interventions must be performed, require more counseling, communication, and consent than situations without interventions. This provides more opportunity for these to go awry, including opportunity for emotional pressure or unfriendly behaviour. Interventions also tend to be more stressful and involve more physical contact. But an explanation is not the same as an excuse. Asking consent prior to a procedure is required by ethics and law [42–44]. Downe et al. (2018) found that in case of a medical intervention, women preferred active decision making to have a sense of control and personal achievement [45]. This underlines the need for focused action on improving the quality of counselling, guidance, and consent in the challenging situation of a peri-partum medical intervention. Our results also show that, in most cases, planned cesarean section did not significantly increase the odds of experiencing upsetting D&A, compared to spontaneous mode of birth. A possible explanation can be that there is more time to focus on women’s experiences in the situation of a non-acute intervention. This may increase the sense of control and autonomy, an important factor for satisfaction of birth [46].

Home birth is already known to have many benefits for low-risk women, including a better overall experience and reduced chances of both iatrogenic and non-iatrogenic injury [47, 48]. Focusing on D&A, compared to giving birth at home, giving birth in a hospital (obstetrician-led) at least doubled the odds of an upsetting experience of D&A in all categories. Birth in an institution (midwife-led) showed increased odds of upsetting experiences of communication issues, lack of support and lack of consent as well. The multivariable model adjusted for interventions of mode of birth and pain relief. However, some interventions like the use of oxytocin augmentation or continuous fetal monitoring with a cardiotocogram (CTG) could not be adjusted for and might provide some explanation [49, 50]. Also, the higher risks of complications during an obstetrician-led hospital birth (as discussed in the previous paragraph) may increase the quantity of interaction, counseling, and need for consent. Perhaps these higher risks might also influence the way healthcare providers interact and the way women experience their birth, although research on this is lacking. Additionally, previous studies found that the environment itself plays a role in birth experiences: women feel more comfortable, free, and in control during a home birth and this contributes to a better experience [30, 46, 51, 52].

It is noteworthy that transfer from midwife-led to obstetrician-led care during birth was associated with upsetting experiences of D&A in almost all categories among both primiparous and multiparous women. The adaptation to a new setting and interruption of the process of labour could impact the sense of autonomy [51, 52]. Most transfers are unplanned and therefore require women to adjust to the new situation and frequently to adjust their birth-wishes [32]. Again, the increased risk for complications in case of a transfer can also impact patient-provider interaction. Additionally, with a transfer of healthcare provider, there is the lack of continuous support, which is associated with more positive experiences of birth [8, 53, 54]. For many categories of D&A, there is no difference in experienced D&A between women who received midwife-led care of obstetrician-led care throughout birth. The increased odds of having experienced upsetting situations of emotional pressure, use of force/physical violence, and lack of support among women who received obstetrician-led care throughout birth might be explained by the organization of maternity care units. This hypothesis is substantiated by research which showed that providers identified factors that drive D&A as: limited staff capacity, high workload, stress and burnout, medical hierarchy, poor facility and lack of supplies [55–58].

Pain relief seems to be an important factor associated with upsetting experiences of D&A. Compared to no use of medical pain relief, all methods of pain relief were associated with more upsetting experiences of D&A in several categories for primiparous women, with the strongest association for lack of support. Whether these experiences were before or after the use of pain relief could not be examined with these data. Women might be more likely to use medical pain relief if they experience lack of support. This is in line with studies on continuity of care which showed fewer women who received continuity of care requested epidural analgesia [59]. It is also possible that healthcare providers assume that women who are receiving pain relief need less support compared to women without pain relief. Unfortunately, research on patient-provider interaction during provision of pain relief is lacking. For multiparous women, only other methods of pain relief were associated with upsetting experiences of unfriendly behaviour/verbal abuse and lack of consent. This suggests that the found associations with D&A are not caused by the methods of pain relief itself, but more likely by the circumstances surrounding it. It is possible that multiparous women who experienced this were too far progressed in labour to receive an epidural or remifentanil, and therefore felt unheard when receiving alternatives. Green & Baston (2003) found that used pethidine or Entonox were associated with lower sense of control of their own behaviour, which may contribute to negative birth experience [46]. To our knowledge, no research on pain relief and experienced D&A has yet been conducted to compare and explain our results.

Previous studies showed that healthcare providers might prevent women’s traumatic birth experiences by better communication, better listening, and giving more (emotional/practical) support [11]. Especially during transfer of care, the use of pain relief, hospital births, and the use of medical interventions, counselling, communication, support, and consent procedures should be improved to help reduce the experience of D&A. Additionally, reorganizing maternity care in such a way that there is more room for continuity of care (even after transfer) and watchful attendance might also help to decrease D&A and improve women’s experiences [60, 61]. Although women who gave birth at home and women without medical interventions report fewer upsetting experiences of D&A compared to women who gave birth in an institution/hospital or underwent medical interventions, this group still shows quite high percentages of communication issues, lack of support and lack of consent. Previous studies showed that most healthcare providers see D&A as a violation of human rights and argue against it, but that they are also aware that D&A is taking place around them [57, 58]. Identifying institutional and personal barriers they experience in providing respectful care is an important area for research and, subsequently, intervention.

### Strengths and limitations

This study is the first to examine associations between birth characteristics and different categories of upsetting D&A in a high-income country. Differences in prevalence of experienced D&A between primiparous and multiparous women confirm the importance of the performed stratification. Due to the high number of respondents, it was possible to look at individual categories of D&A and adjust for many confounders.

Measuring D&A is difficult and a valid instrument to do so was not yet available at the start of this study [62]. In this explorative study, a new questionnaire was developed. This provided not only insight into experiences of D&A, but also into whether women considered this upsetting. Although the questionnaire was not validated, it was developed by a multidisciplinary team, including patients, and tested in multiple rounds. Hereby, the Dutch context was taken into account.

In this study, all data were self-reported. Self-reported data may lead to bias. For example, there is a risk of recall bias since women answered questions about their birth from up to a maximum of five years before. However, there is evidence that, years later, women’s memories of their birth are generally accurate [63]. Although not all women might experience certain situations as D&A, or as upsetting, it is the woman’s personal experience of D&A that we want to understand and gain insight into. Therefore, self-reported data are appropriate in this study. Because of the retrospective data, the found associations cannot simply be interpret as causal connections. This must be kept in mind whilst interpreting our results. Further research is necessary to provide more insight into the causality of our found associations, and to examine why women experience certain situations as (upsetting) D&A.

Even though we emphasized that all experiences matter, women with (very) negative experiences might have been more likely to participate. However, it is notable that the number of home births is higher than the national average [64]. With the lower odds of experienced D&A among women who gave birth at home, the actual prevalence of D&A might even be higher. Nevertheless, the prevalence of negative birth experience corresponds to data from other high-income countries [5–7, 17].

As with other studies, women with non-Dutch ethnic background and low level of education were underrepresented compared to national statistics [17, 65, 66]. This might be due to the use of social media as main recruitment method, and the language options being English and Dutch only. Numbers of upsetting experienced discrimination were too small to calculate AORs and CIs of the univariable analyses were wide. Since people with non-Dutch ethnic background are more often discriminated, and were underrepresented in this study, these numbers might not be representative. Research with a more representative sample is necessary to examine which birth characteristics are associated with discrimination.

### Implications for research and clinical practice

This study provides insight into how often women experience D&A during birth (as upsetting) and which birth characteristics are associated with this. To help understand why D&A occurs more often in specific situations, and why women experience situations upsetting, qualitative studies should be performed. Also, for better understanding and to help formulate targeted recommendations for training professionals and improving institutions, further research focussing on the provider’s perspective regarding D&A during birth is necessary. Additionally, further studies should include hard to reach groups for a more representative study population.

For healthcare providers caring for women during birth, it is important to be aware that certain situations can be experienced as D&A, and to realize that they themselves have an impact on women’s experiences [17]. Especially in case of transfer of care, hospital births and situations of unplanned interventions, it is important to focus on adequate verbal and non-verbal guidance, support, counseling, and consent. For the guidance of primiparous women, there must be extra attention for dealing with pain and the use of medical pain relief. Additionally, adjusting the maternity care system in such a way that there is less work pressure and more time for continuity of care and watchful attendance can contribute to lower rates of experienced D&A. The decrease of upsetting experiences of D&A and thereby of negative birth experiences will improve quality of care [1].

## Conclusions

The experience of at least one upsetting situation of D&A during birth was reported by 45.1% of primiparous and 27.0% of multiparous women. Especially transfer from midwife-led to obstetrician-led care, giving birth in a hospital, the use of (unplanned) interventions, and the use of medical pain relief were factors increasing the odds of women experiencing D&A. The high prevalence of D&A during birth emphasizes the need for more attention to verbal and non-verbal communication, support and adequate decision-making and consent procedures, especially during the situations of birth that are associated with D&A. Having a positive birth experience is an important aspect of good quality maternity care, regardless of circumstances.

### Supplementary Information

**Supplementary Material 1.**

**Supplementary Material 2.**

**Supplementary Material 3.**

## Data Availability

The dataset used during the current study is available from the corresponding author on reasonable request.

## Abbreviations

D&A: Disrespect and abuse
WHO: World Health Organization
MREC: Medical Research Ethics Committee
UMC: University medical center
IP: Internet protocol
IT: Information technology
CBS: Central Bureau of Statistics
C-section: Caesarean section
VIF: Variance inflation factor
OR: Odds ratio
AOR: Adjusted odds ratio
CI: Confidence interval
SPSS: Statistical Package for the Social Sciences
CTG: Cardiotocogram
