# Supplementary material for: Experienced disrespect & abuse during childbirth and associated birth characteristics: a cross-sectional survey in the Netherlands
Source: BMC Pregnancy Childbirth. 2024 Feb 29;24:170. doi: 10.1186/s12884-024-06360-y (PMC10905902; doi:10.1186/s12884-024-06360-y)
Supplement: Supplementary file 1 — Supplementary Material 1. [file 12884_2024_6360_MOESM1_ESM.docx]

# Overview of the questions regarding D&A

Questions were divided into seven categories with answer options yes/no. If they had a negative experience, women received two follow-up questions: ‘Have you experienced this as upsetting?’[yes/no] and ‘if you want to share something about this experience, you can do so here’ [open question].

| **Emotional pressure** |
| --- |
| Were you threatened with bad test results or poor outcomes related to the health of your child? Were threats made that involved withholding care from you or your child? Were you threatened with legal consequences (for example: a child protective services report, criminal charges or other legal proceedings?) |
| **Unfriendly behaviour/verbal abuse** |
| Did a health care provider say you were overreacting or you were pretending things were worse than they really were? Were you subjected to insulting, harsh, unpleasant and/or derogatory comments? Were you spoken to or shouted at in a harsh/rough or crude/coarse way? Were you verbally abused? |
| **Use of force/physical violence** |
| Were you forced to stay in bed? Were you forced into a particular position, or were you manually restrained? Were you subject to rough physical treatment? (For example: pushing, pulling, pinching/gripping, pushing your legs in a particular position). Were you slapped or kicked? Was a (medical) intervention performed that you experienced as physical abuse? Was a (medical) intervention performed that you experienced as sexual abuse? |
| **Communication issues** |
| Did you feel you were not being involved in the decision-making during labour and birth? Did you feel you were not being listened to? Did you feel you were not being taken seriously? Did you feel insufficiently at ease to ask questions? Did you feel that you weren’t being given information that you should have been given? (For example: not being given full information about what was going on; not being informed about risks and benefits; non-disclosure of test results or diagnoses; not being given information about the progress of labour, not being provided with alternative options). |
| **Lack of support** |
| Did you feel you received too little attention, or were you left alone when you did not want to be left alone? Did a health care provider refuse to assist you? (For example: assistance with going to the bathroom; help with taking a shower; help with managing contractions). Did you ask for pain relief and was your request either ignored or refused by the care provider without there being a clear reason for this? (For example: pain relief during labour or a local anaesthetic during suturing). Were you or your partner denied (physical) contact with your child, without a clear reason? Did you experience a lack of privacy? (For example: during a physical examination). |
| **Lack of consent** |
| Were you not free to decide who would be present at your delivery (other than health care providers)? Did you feel compelled to accept care that you didn’t really want? Were you not free to decide your position during contractions? Were you not free to decide the position in which you gave birth? Were you told there were certain things you weren’t allowed to do, without there being a clear reason for this decision? (For example: not being allowed to make noise, no eating, drinking, walking around, taking a shower). Was a (medical) intervention done without your having given clear permission in advance? (For example: a vaginal examination, breaking your waters, performing an episiotomy; administering an injection after the birth). Was a (medical) intervention continued even after you asked for it to be stopped? (For example: being restrained during a vaginal examination). |
| **Discrimination** |
| Did you experience discrimination based on race, ethnicity, cultural background, or language? Did you experience discrimination based on age? Did you experience discrimination based on sexuality and/or gender identity? Did you experience discrimination based on physical or mental disability, illness or complaint? Did you experience discrimination based on religion or belief? Did you experience discrimination based on your appearance (other than racial appearance)? Did you experience discrimination based on education, class, income or other socio-economic factors? |
