# Supplementary material for: Experienced disrespect & abuse during childbirth and associated birth characteristics: a cross-sectional survey in the Netherlands
Source: BMC Pregnancy Childbirth. 2024 Feb 29;24:170. doi: 10.1186/s12884-024-06360-y (PMC10905902; doi:10.1186/s12884-024-06360-y)
Supplement: Supplementary file 2 — Supplementary Material 2. [file 12884_2024_6360_MOESM2_ESM.docx]

# Categorization of baseline and birth characteristics

| **Characteristic** | **Categories** | **Explanation** |
| --- | --- | --- |
| **Maternal age** | <25 years | <25 years at the time of birth |
|  | 25-35 years | 25-35 years at the time of birth |
|  | >35 years | >35 years at the time of birth |
| **Ethnic background** | Dutch | Participants and both parents are born in the Netherlands |
|  | Western | Participant or at least one of the parents is born in a Western country |
|  | Non-western | Participant or at least one of the parents is born in a non-Western country |
| **Level of education** | Low | Primary school or preparatory vocational secundary education (VMBO) |
|  | Middle | Vocational education and training (MBO), senior general secondary education (HAVO), and university preparatory education (VWO) |
|  | High | Higher professional education (HBO), and research-oriented education (WO) |
| **Marital Status at time of birth** | Married | Married |
|  | Living together | Living together, but not married |
|  | Living apart together | In a relationship, but not living together |
|  | Single | Not in a relationship |
| **Singleton or multiple pregnancy** | Singleton | Pregnant with one child |
|  | Multiple | Pregnant with more than one child |
| **Gestational age** | Preterm | <37 weeks |
|  | Term | 37-41+6 weeks |
|  | Postterm | ≥42 weeks |
| **Before or during COVID-19 pandemic** | Before COVID-19 pandemic | 2015 – February 2020 |
|  | During COVID-19 pandemic | March – December 2020 |
| **Onset of labour** | Spontaneous | Start with spontaneous rupture of the membranes or spontaneous contractions |
|  | Induction | Start labour with a catheter, artificial rupture of the membranes or medication |
|  | C-section | Planned cesarean section, before labour started spontaneously |
| **Healthcare provider throughout birth** | Midwife-led care | Care from the community midwife during the whole proces of labour |
|  | Transfer | Transfer of healthcare provider during birth from midwife-led care to obstetrician-led care |
|  | Obstetrician-led care | Care from a hospital-based care provider during the whole proces of labour |
| **Pain relief during labour** | None | No use of medical pain relief during labour |
|  | Epidural analgesia | Use of epidural analgesia during labour |
|  | Remifentanil | Use of remifentanil during labour |
|  | Other | Use of sterile water injection, Entonox, or pethidine during labour |
| **Place of birth** | Home | Home |
|  | Midwife-led institutional | Birth centre or hospital with community midwife |
|  | Obstetrician-led hospital | Hospital with hospital-based care provider (medical indication) |
| **Mode of birth** | Spontaneous | Spontaneous vaginal birth without interventions |
|  | Planned C-section | Cesarean section, decided during the pregnancy |
|  | Spontaneous with episiotomy | Spontaneous vaginal birth after episiotomy (only) |
|  | Assisted vaginal birth | Vaginal birth with help of vacuum or forceps |
|  | Unplanned C-section | Cesarean section, decided during birth |
