# Supplementary material for: Experienced disrespect & abuse during childbirth and associated birth characteristics: a cross-sectional survey in the Netherlands
Source: BMC Pregnancy Childbirth. 2024 Feb 29;24:170. doi: 10.1186/s12884-024-06360-y (PMC10905902; doi:10.1186/s12884-024-06360-y)
Supplement: Supplementary file 3 — Supplementary Material 3. [file 12884_2024_6360_MOESM3_ESM.docx]

|  | | **Any category of disrespect and abuse** | | **Emotional pressure** | | **Unfriendly behaviour/**  **verbal abuse** | | **Use of force / physical abuse** | | **Communication issues** | | **Lack of support** | | **Lack of consent** | | **Discrimination** | |
| --- | --- | --- | --- | --- | --- | --- | --- | --- | --- | --- | --- | --- | --- | --- | --- | --- | --- |
|  |  | Not experienced or upsetting  n (%) | Upsetting  n (%) | Not experienced  or upsetting  n (%) | Upsetting  n (%) | Not experienced  or upsetting  n (%) | Upsetting  n (%) | Not experienced  or upsetting  n (%) | Upsetting  n (%) | Not experienced  or upsetting  n (%) | Upsetting  n (%) | Not experienced  or upsetting  n (%) | Upsetting  n (%) | Not experienced  or upsetting  n (%) | Upsetting  n (%) | Not experienced  or upsetting  n (%) | Upsetting  n (%) |
| **Total** | | 3633 (54.9) | 2983 (45.1) | 6411 (96.9) | 205 (3.1) | 5945 (89.9) | 671 (10.1) | 5578 (84.3) | 1038 (15.7) | 4863 (73.5) | 1753 (26.5) | 5113 (77.3) | 1503 (22.7) | 4851 (73.3) | 1765 (26.7) | 6556 (99.1) | 60 (0.9) |
| **Onset of labour** | Spontaneous | 2599 (59.3) | 1784 (40.7) | 4266 (97.3) | 117 (2.7) | 3982 (90.9) | 401 (9.1) | 3775 (86.1) | 608 (13.9) | 3327 (75.9) | 1056 (24.1) | 3483 (79.5) | 900 (20.5) | 3349 (76.4) | 1034 (23.6) | 4352 (99.3) | 31 (0.7) |
|  | Induction | 878 (44.8) | 1082 (55.2) | 1880 (95.9) | 80 (4.1) | 1717 (87.6) | 243 (12.4) | 1552 (79.2) | 408 (20.8) | 1326 (67.7) | 634 (32.3) | 1428 (72.9) | 532 (27.1) | 1276 (65.1) | 684 (34.9) | 1935 (98.7) | 25 (1.3) |
|  | C-section | 156 (57.1) | 117 (42.9) | 265 (97.1) | 8 (2.9) | 246 (90.1) | 27 (9.9) | 251 (91.9) | 22 (8.1) | 210 (76.9) | 63 (23.1) | 202 (74.0) | 71 (26.0) | 226 (82.8) | 47 (17.2) | 269 (98.5) | 4 (1.5) |
| **Healthcare provider throughout birth** | Midwife-led care | 1255 (80.6) | 303 (19.4) | 1542 (99.0) | 16 (1.0) | 1513 (97.1) | 45 (3.9) | 1483 (95.2) | 75 (4.8) | 1413 (90.7) | 145 (9.3) | 1427 (91.6) | 131 (8.4) | 1403 (90.1) | 155 (9.9) | 1557 (99.9) | 1 (0.1) |
|  | Transfer of care° | 1141 (47.1) | 1281 (52.9) | 2341 (96.7) | 81 (3.3) | 2117 (87.4) | 305 (12.6) | 1953 (80.6) | 469 (19.4) | 1631 (67.3) | 791 (32.7) | 1770 (73.1) | 652 (26.9) | 1643 (67.8) | 779 (32.2) | 2399 (99.1) | 23 (0.9) |
|  | Obstetrician-led care | 1237 (46.9) | 1399 (53.1) | 2528 (95.9) | 108 (4.1) | 2315 (87.8) | 321 (12.2) | 2142 (81.3) | 494 (18.7) | 1819 (69.0) | 817 (31.0) | 1916 (72.7) | 720 (27.3) | 1805 (68.5) | 831 (31.5) | 2600 (98.6) | 36 (1.4) |
| **Pain relief during labour** | None | 2164 (65.7) | 1132 (34.3) | 3217 (97.6) | 79 (2.4) | 3074 (93.3) | 222 (6.7) | 2914 (88.4) | 382 (11.6) | 2675 (81.2) | 621 (18.8) | 2757 (83.6) | 539 (16.4) | 2628 (79.7) | 668 (20.3) | 3280 (99.5) | 16 (0.5) |
|  | Epidural | 870 (43.1) | 1147 (56.9) | 1938 (96.1) | 79 (3.9) | 1727 (85.6) | 290 (14.4) | 1617 (80.2) | 400 (19.8) | 1316 (65.2) | 701 (34.8) | 1430 (70.9) | 587 (29.1) | 1316 (65.2) | 701 (34.8) | 1985 (98.4) | 32 (1.6) |
|  | Remifentanil | 336 (41.3) | 477 (58.7) | 779 (95.8) | 34 (4.2) | 709 (87.2) | 104 (12.8) | 615 (75.6) | 198 (24.4) | 518 (63.7) | 295 (36.3) | 573 (70.5) | 240 (29.5) | 512 (63.0) | 301 (37.0) | 806 (99.1) | 7 (0.9) |
|  | Other^ | 84 (45.9) | 99 (54.1) | 179 (97.8) | 4 (2.2) | 157 (85.8) | 26 (14.2) | 149 (81.4) | 34 (18.6) | 117 (63.9) | 66 (36.1) | 122 (66.7) | 61 (33.3) | 136 (74.3) | 47 (25.7) | 182 (99.5) | 1 (0.5) |
| **Place of birth** | Home | 738 (85.0) | 130 (15.0) | 860 (99.1) | 8 (0.9) | 850 (97.9) | 18 (2.1) | 829 (95.5) | 39 (4.5) | 813 (93.7) | 55 (6.3) | 820 (94.5) | 48 (5.5) | 805 (92.7) | 63 (7.3) | 867 (99.9) | 1 (0.1) |
|  | Midwife-led institutional* | 636 (75.4) | 208 (24.6) | 831 (98.5) | 13 (1.5) | 814 (96.4) | 30 (3.6) | 795 (94.2) | 49 (5.8) | 736 (87.2) | 108 (12.8) | 747 (88.5) | 97 (11.5) | 739 (87.6) | 105 (12.4) | 842 (99.8) | 2 (0.2) |
|  | Obstetrician-led hospital** | 2259 (46.1) | 2645 (53.9) | 4720 (96.2) | 184 (3.8) | 4281 (87.3) | 623 (12.7) | 3954 (80.6) | 950 (19.4) | 3314 (67.6) | 1590 (32.4) | 3546 (72.3) | 1358 (27.7) | 3307 (67.4) | 1597 (32.6) | 4847 (98.8) | 57 (1.2) |
| **Mode of birth** | Spontaneous | 2223 (66.0) | 1145 (34.0) | 3282 (97.4) | 86 (2.6) | 3135 (93.1) | 233 (6.9) | 3056 (90.7) | 312 (9.3) | 2768 (82.2) | 600 (17.8) | 2813 (83.5) | 555 (16.5) | 2739 (81.3) | 629 (18.7) | 3349 (99.4) | 19 (0.6) |
|  | Planned  C-section | 148 (62.4) | 89 (37.6) | 230 (97.0) | 7 (3.0) | 217 (91.6) | 20 (8.4) | 222 (93.7) | 15 (6.3) | 189 (79.7) | 48 (20.3) | 182 (76.8) | 55 (23.2) | 206 (86.9) | 31 (13.1) | 234 (98.7) | 3 (1.3) |
|  | Spontaneous with episiotomy | 535 (50.7) | 523 (49.4) | 1020 (96.4) | 38 (3.6) | 939 (88.8) | 119 (11.2) | 851 (80.4) | 207 (19.6) | 756 (71.5) | 302 (28.5) | 803 (75.9) | 255 (24.1) | 731 (69.1) | 327 (30.9) | 1043 (98.6) | 15 (1.4) |
|  | Assisted vaginal birth | 375 (38.9) | 590 (61.1) | 939 (97.3) | 26 (2.7) | 825 (85.5) | 140 (14.5) | 693 (71.8) | 272 (28.2) | 596 (61.8) | 369 (38.2) | 670 (69.4) | 295 (30.6) | 576 (59.7) | 389 (40.3) | 958 (99.3) | 7 (0.7) |
|  | Unplanned  C-section | 352 (35.6) | 636 (64.4) | 940 (95.1) | 48 (4.9) | 829 (83.9) | 159 (16.1) | 756 (76.5) | 232 (23.5) | 554 (56.1) | 434 (43.9) | 645 (65.3) | 343 (34.7) | 599 (60.6) | 389 (39.4) | 972 (98.4) | 16 (1.6) |

*Table 5: Descriptive statistics of upsetting experiences of disrespect and abuse for all birth characteristics among primiparous women (n=6,616)*

°Transfer from midwife-led care to obstetrician-led care, ^Sterile water injection, Entonox or pethidine, *Birth centre or hospital with community midwife, **Hospital with hospital-based care provider (medical indication), C-section: caesarean section.

|  | | **Any category of disrespect and abuse** | | **Emotional pressure** | | **Unfriendly behaviour/**  **verbal abuse** | | **Use of force / physical abuse** | | **Communication issues** | | **Lack of support** | | **Lack of consent** | | **Discrimination** | |
| --- | --- | --- | --- | --- | --- | --- | --- | --- | --- | --- | --- | --- | --- | --- | --- | --- | --- |
|  |  | Not experienced or upsetting  n (%) | Upsetting  n (%) | Not experienced  or upsetting  n (%) | Upsetting  n (%) | Not experienced  or upsetting  n (%) | Upsetting  n (%) | Not experienced  or upsetting  n (%) | Upsetting  n (%) | Not experienced  or upsetting  n (%) | Upsetting  n (%) | Not experienced  or upsetting  n (%) | Upsetting  n (%) | Not experienced  or upsetting  n (%) | Upsetting  n (%) | Not experienced  or upsetting  n (%) | Upsetting  n (%) |
| **Total** | | 3581 (73.0) | 1323 (27.0) | 4808 (98.0) | 96 (2.0) | 4625 (94.3) | 279 (5.7) | 4461 (91.0) | 443 (9.0) | 4235 (86.4) | 669 (13.6) | 4317 (88.0) | 587 (12.0) | 4163 (84.9) | 741 (15.1) | 4881 (99.5) | 23 (0.5) |
| **Onset of labour** | Spontaneous | 2627 (77.8) | 749 (22.2) | 3314 (98.2) | 62 (1.8) | 3225 (95.5) | 151 (4.5) | 3117 (92.3) | 259 (7.7) | 3011 (89.2) | 365 (10.8) | 3064 (90.8) | 312 (9.2) | 2946 (87.3) | 430 (12.7) | 3365 (99.7) | 11 (0.3) |
|  | Induction | 697 (60.7) | 451 (39.3) | 1119 (97.5) | 29 (2.5) | 1047 (91.2) | 101 (8.8) | 994 (86.6) | 154 (13.4) | 919 (80.1) | 229 (19.9) | 939 (81.8) | 209 (18.2) | 870 (75.8) | 278 (24.2) | 1141 (99.4) | 7 (0.6) |
|  | C-section | 257 (67.6) | 123 (32.4) | 375 (98.7) | 5 (1.3) | 353 (92.9) | 27 (7.1) | 350 (92.1) | 30 (7.9) | 305 (80.3) | 75 (19.7) | 314 (82.6) | 66 (17.4) | 347 (91.3) | 33 (8.7) | 375 (98.7) | 5 (1.3) |
| **Healthcare provider throughout birth** | Midwife-led care | 2016 (85.1) | 325 (13.9) | 2320 (99.1) | 21 (0.9) | 2297 (98.1) | 44 (1.9) | 2263 (96.7) | 78 (3.3) | 2211 (94.4) | 130 (5.6) | 2220 (94.8) | 121 (5.2) | 2168 (92.6) | 173 (7.4) | 2337 (99.8) | 4 (0.2) |
|  | Transfer of care° | 410 (61.4) | 258 (38.6) | 638 (95.5) | 30 (4.5) | 605 (90.6) | 63 (9.4) | 556 (83.2) | 112 (16.8) | 521 (78.0) | 147 (22.0) | 556 (83.2) | 112 (16.8) | 499 (74.7) | 169 (25.3) | 665 (99.6) | 3 (0.4) |
|  | Obstetrician-led care | 1155 (60.9) | 740 (39.1) | 1850 (97.6) | 45 (2.4) | 1723 (90.9) | 172 (9.1) | 1642 (86.6) | 253 (13.4) | 1503 (79.3) | 392 (20.7) | 1541 (81.3) | 354 (18.7) | 1496 (78.9) | 399 (21.1) | 1879 (99.2) | 16 (0.8) |
| **Pain relief during labour** | None | 2829 (77.5) | 821 (22.5) | 3588 (98.3) | 62 (1.7) | 3502 (95.9) | 148 (4.1) | 3387 (92.8) | 263 (7.2) | 3261 (89.3) | 389 (10.7) | 3314 (90.8) | 336 (9.2) | 3172 (86.9) | 478 (13.1) | 3639 (99.7) | 11 (0.3) |
|  | Epidural | 222 (54.4) | 186 (45.6) | 396 (97.1) | 12 (2.9) | 360 (88.2) | 48 (11.8) | 341 (83.6) | 67 (16.4) | 312 (76.5) | 96 (23.5) | 321 (78.7) | 87 (21.3) | 298 (73.0) | 110 (27.0) | 405 (99.3) | 3 (0.7) |
|  | Remifentanil | 223 (60.1) | 148 (39.9) | 358 (96.5) | 13 (3.5) | 331 (89.2) | 40 (10.8) | 302 (81.4) | 69 (18.6) | 284 (76.5) | 87 (23.5) | 294 (79.2) | 77 (20.8) | 281 (75.7) | 90 (24.3) | 368 (99.2) | 3 (0.8) |
|  | Other^ | 35 (47.9) | 38 (52.1) | 69 (94.5) | 4 (5.5) | 60 (82.2) | 13 (17.8) | 59 (80.8) | 14 (19.2) | 54 (74.0) | 19 (26.0) | 56 (76.7) | 17 (23.3) | 46 (63.0) | 27 (37.0) | 72 (98.6) | 1 (1.4) |
| **Place of birth** | Home | 1345 (87.8) | 187 (12.2) | 1520 (99.2) | 12 (0.8) | 1510 (98.6) | 22 (1.4) | 1483 (96.8) | 49 (3.2) | 1457 (95.1) | 75 (4.9) | 1468 (95.8) | 64 (4.2) | 1432 (93.5) | 100 (6.5) | 1530 (99.9) | 2 (0.1) |
|  | Midwife-led institutional* | 782 (82.1) | 170 (17.9) | 945 (99.3) | 7 (0.7) | 928 (97.5) | 24 (2.5) | 915 (96.1) | 37 (3.9) | 889 (93.4) | 63 (6.6) | 876 (92.0) | 76 (8.0) | 863 (90.7) | 89 (9.3) | 950 (99.8) | 2 (0.2) |
|  | Obstetrician-led hospital** | 1454 (60.1) | 966 (39.9) | 2343 (96.8) | 77 (3.2) | 2187 (90.4) | 233 (9.6) | 2063 (85.2) | 357 (14.8) | 1889 (78.1) | 531 (21.9) | 1973 (81.5) | 447 (18.5) | 1868 (77.2) | 552 (22.8) | 2401 (99.2) | 19 (0.8) |
| **Mode of birth** | Spontaneous | 2978 (76.8) | 902 (23.2) | 3811 (98.2) | 69 (1.8) | 3704 (95.5) | 176 (4.5) | 3590 (92.5) | 290 (7.5) | 3463 (89.3) | 417 (10.7) | 3492 (90.0) | 388 (10.0) | 3364 (86.7) | 516 (13.3) | 3867 (99.7) | 13 (0.3) |
|  | Planned  C-section | 247 (70.0) | 106 (30.0) | 350 (99.2) | 3 (0.8) | 331 (93.8) | 22 (6.2) | 327 (92.6) | 26 (7.4) | 292 (82.7) | 61 (17.3) | 295 (83.6) | 58 (16.4) | 324 (91.8) | 29 (8.2) | 349 (98.9) | 4 (1.1) |
|  | Spontaneous with episiotomy | 209 (67.2) | 102 (32.8) | 305 (98.1) | 6 (1.9) | 293 (94.2) | 18 (5.8) | 281 (90.4) | 30 (9.6) | 263 (84.6) | 48 (15.4) | 270 (86.8) | 41 (13.2) | 250 (80.4) | 61 (19.6) | 311 (100.0) | 0 (0.0) |
|  | Assisted vaginal birth | 39 (38.6) | 62 (61.4) | 96 (95.0) | 5 (5.0) | 82 (81.2) | 19 (18.8) | 70 (69.3) | 31 (30.7) | 64 (63.4) | 37 (36.6) | 78 (77.2) | 23 (22.8) | 58 (57.4) | 43 (42.6) | 100 (99.0) | 1 (1.0) |
|  | Unplanned  C-section | 108 (41.7) | 151 (58.3) | 246 (95.0) | 13 (5.0) | 215 (83.0) | 44 (17.0) | 193 (74.5) | 66 (25.5) | 153 (59.1) | 106 (40.9) | 182 (70.3) | 77 (29.7) | 167 (64.5) | 92 (35.5) | 245 (98.1) | 5 (1.9) |

*Table 6: Descriptive statistics of upsetting experiences of disrespect and abuse for all birth characteristics among multiparous women (n=4,904)*

°Transfer from midwife-led care to obstetrician-led care, ^Sterile water injection, Entonox or pethidine, *Birth centre or hospital with community midwife, **Hospital with hospital-based care provider (medical indication), C-section: caesarean section.
